# Strangulated Small Bowel Within a Posterior Rectus Sheath Hernia in a Patient Without Prior Abdominal Surgery

**DOI:** 10.1155/cris/9048989

**Published:** 2026-08-04

**Authors:** Rebecca Deitch, Megan Kolbe, Patrick D. Melmer

**Affiliations:** ^1^ Department of Surgery, Virginia Commonwealth University, Richmond, Virginia, USA, vcu.edu; ^2^ Department of Surgery, Emory University, Atlanta, Georgia, USA, emory.edu

**Keywords:** acute abdomen, interparietal hernia, posterior rectus sheath hernia, small bowel obstruction, strangulated hernia

## Abstract

**Background:**

Posterior rectus sheath hernia is an exceptionally rare interparietal abdominal wall hernia in which abdominal contents herniate through the posterior rectus sheath into the retrorectus space while the anterior rectus sheath remains intact. Clinical diagnosis may be difficult because the hernia remains confined within the abdominal wall, and delayed recognition may result in bowel strangulation. We report a rare case of spontaneous posterior rectus sheath hernia complicated by bowel necrosis requiring staged operative management.

**Case Presentation:**

A 73‐year‐old woman with no history of previous abdominal surgery presented with a 48‐h history of progressively worsening epigastric abdominal pain, nausea, and emesis. Computed tomography demonstrated a narrow‐neck posterior rectus sheath hernia containing incarcerated small bowel with decreased bowel wall enhancement, proximal small bowel dilatation, and findings concerning for strangulation. Emergency exploratory laparotomy confirmed an approximately 1‐cm posterior rectus sheath defect with incarcerated mid‐jejunum within the retrorectus space. Following reduction, a 10‐cm segment of necrotic jejunum with pinpoint perforation required resection. Because adjacent bowel viability remained uncertain, temporary abdominal closure with negative‐pressure therapy was performed followed by a planned second‐look laparotomy 24 h later. Re‐exploration demonstrated complete recovery of the remaining bowel, allowing stapled side‐to‐side functional end‐to‐end small bowel anastomosis and primary repair of the posterior rectus sheath without mesh because of contamination. The patient experienced an uncomplicated postoperative recovery, was discharged home on postoperative day five, and remained free of recurrent posterior rectus sheath hernia or incisional hernia at 1‐month clinical follow‐up and 1‐year computed tomography surveillance.

**Conclusion:**

Posterior rectus sheath hernia should be considered in patients presenting with small bowel obstruction and an interparietal abdominal wall defect on computed tomography, even in the absence of previous abdominal surgery. Careful recognition of the characteristic retrorectus anatomy and prompt operative intervention are essential when strangulation is suspected. This case demonstrates successful staged management with bowel resection, planned second‐look laparotomy, and durable primary repair with no recurrence at 1 year.

## 1. Introduction

Posterior rectus sheath hernia is an exceptionally uncommon abdominal wall hernia that has been reported following prior abdominal surgery, abdominal wall trauma, rectus sheath hematoma, or, less commonly, as a spontaneous lesion. Unlike conventional ventral or incisional hernias, these entities are classified as interparietal hernias because the herniated viscera remain confined between layers of the abdominal wall rather than extending into the subcutaneous tissues. Herniation occurs through a defect in the posterior rectus sheath into the retrorectus space, while the anterior rectus sheath remains intact, producing a deep abdominal wall hernia that may be difficult to recognize clinically. Interparietal hernias are traditionally classified into preperitoneal, interstitial, and superficial subtypes based on the anatomical plane occupied by the hernia sac. Posterior rectus sheath hernia represents an uncommon interstitial variant in which the bowel becomes trapped within the rectus compartment posterior to the rectus abdominis muscle [1, 2].

A limited number of cases of posterior rectus sheath hernia have been reported in the literature. Reported etiologies include rectus sheath hematoma, cesarean section, disruption of the posterior sheath following enhanced‐view totally extraperitoneal ventral hernia repair, and other previous abdominal operations, although spontaneous cases without prior abdominal surgery remain exceptionally uncommon [2–5]. If incidentally found and asymptomatic, they may be observed [6]. We present a rare case of spontaneous posterior rectus sheath hernia resulting in strangulated small bowel requiring bowel resection and planned second‐look laparotomy in a patient without prior abdominal surgery. Written informed consent for the publication of this case report and accompanying images was obtained from the patient.

## 2. Presentation of Case

**Figure 1:**
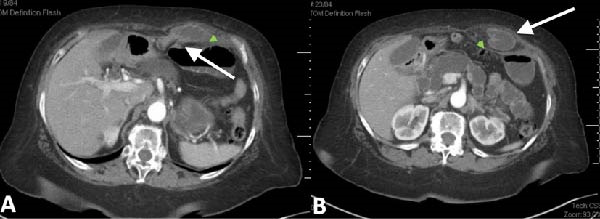
(A) CT images depicting neck defect in the posterior rectus sheath (white arrow) with surrounding rectus muscle (green arrowhead). (B) Herniated bowel (white arrow) through the posterior rectus sheath (green arrowhead).

A 73‐year‐old woman presented to the emergency department with approximately 48 h of progressively worsening epigastric abdominal pain associated with nausea and repeated episodes of emesis. She denied having previous abdominal operations. Her past medical history was significant for atrial fibrillation, for which she was receiving chronic apixaban therapy, and hypothyroidism being treated with levothyroxine. Her BMI was 25 kg/m^2^. No identifiable predisposing factors for spontaneous abdominal wall hernia formation were identified, specifically a history of chronic cough, constipation, previous abdominal trauma, or rectus sheath hematoma. Vital signs demonstrated a systolic blood pressure of 154/78, heart rate of 78, respirations of 18, and 95% O_2_ saturation on room air. Physical examination demonstrated localized guarding over the left upper paramedian abdomen with a nonreducible abdominal wall mass. Laboratory evaluation demonstrated a normal white blood cell count and normal serum lactate concentration. Computed tomography imaging demonstrated a narrow neck defect within the left paramedian rectus sheath between the linea alba and linea semilunaris, suggestive of a posterior rectus sheath hernia as well as proximal bowel dilation and distal decompression. The defect contained a loop of bowel with decreased bowel wall enhancement concerning for incarceration and strangulation with vascular compromise (Figure 1). The bowel remained confined within the posterior rectus compartment, distinguishing the lesion from a Spigelian hernia, which characteristically occurs through the Spigelian fascia along the linea semilunaris. Given the concern for bowel strangulation, the patient underwent urgent operative exploration.

**Figure 2:**
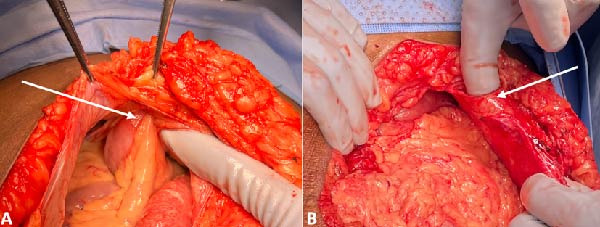
(A) Intraoperative image depicting herniated small bowel through the posterior rectus sheath (white arrow). (B) Surgeon’s finger inside rectus sheath defect (white arrow).

A midline laparotomy was performed. Exploration demonstrated an approximately 1‐cm posterior rectus sheath defect just lateral to the linea alba, medial to the semilunaris, and superior to the arcuate line, concordant with imaging, confirming that it was not a Spigelian hernia defect (Figure 2). The anterior rectus sheath remained completely intact. Through this defect, an incarcerated loop of small bowel had entered the retrorectus space and become tightly strangulated. The fascial defect was carefully enlarged to permit an atraumatic reduction of the incarcerated bowel. A 10‐cm segment of mid‐jejunum demonstrated irreversible ischemic necrosis with a pinpoint perforation, necessitating bowel resection. Because the viability of the adjacent bowel remained uncertain, temporary abdominal closure using a negative‐pressure vacuum dressing was performed with plans for a second‐look laparotomy 24 h later. The patient returned to the operating room 24 h later, where re‐exploration demonstrated complete recovery of the remaining bowel with no further ischemia. A stapled side‐to‐side functional end‐to‐end small bowel anastomosis was performed, and the posterior rectus sheath defect was repaired primarily with running 3–0 polydioxanone suture incorporating the peritoneum as well after excision of the hernia sac. The mesh was not utilized due to the perforation and contamination present at the index operation. The abdominal wall was then closed in a standard fashion.

The patient’s postoperative course was uncomplicated. She had a return of bowel function on postoperative day 3 and was discharged to home on postoperative day 5, tolerating a regular diet. At 1 month follow‐up, she remained well without evidence of recurrent posterior rectus sheath hernia or incisional hernia. Subsequent CT imaging performed at 1 year demonstrated the same, with no evidence of recurrent posterior rectus sheath hernia or incisional hernia.

## 3. Discussion

Posterior rectus sheath hernia is an exceptionally uncommon form of interparietal abdominal wall hernia in which abdominal contents herniate through a defect in the posterior rectus sheath into the retrorectus space, while the anterior rectus sheath remains intact. Because the hernia remains confined between layers of the abdominal wall rather than extending into the subcutaneous tissues, clinical diagnosis may be challenging, and the condition is frequently identified only with cross‐sectional imaging or at operative exploration [1, 2]. Although the true incidence is unknown, posterior rectus sheath hernia remains one of the rarest abdominal wall hernias described in the literature.

The anatomy of the posterior rectus sheath is central to understanding this uncommon pathology. Superior to the arcuate line, the posterior rectus sheath is formed primarily by the posterior lamina of the internal oblique aponeurosis, together with the transversus abdominis aponeurosis. Inferior to the arcuate line, all three lateral abdominal wall aponeuroses pass anterior to the rectus muscle, leaving only the transversalis fascia and peritoneum posteriorly. Consequently, most reported posterior rectus sheath hernias occur above the arcuate line where a true posterior rectus sheath exists, although isolated cases inferior to the arcuate line have been described [2, 7].

The diagnosis should also be distinguished from other interparietal abdominal wall hernias, particularly Spigelian hernia. Spigelian hernias arise through a defect in the Spigelian fascia along the linea semilunaris, whereas posterior rectus sheath hernias occur through the posterior rectus sheath with herniation into the retrorectus space and preservation of the anterior rectus sheath. In the present case, operative exploration confirmed an intact anterior rectus sheath with an incarcerated small bowel located entirely within the retrorectus compartment, confirming the diagnosis of posterior rectus sheath hernia rather than Spigelian hernia. Diagnosis relies on maintaining clinical suspicion together with careful interpretation of cross‐sectional imaging. A history of recent abdominal surgery, especially surgeries requiring abdominal incisions overlying the rectus sheath, is an important diagnostic clue. The patient may present with symptoms, such as nausea, vomiting, and poorly localized abdominal pain, although some case reports state that patients presented asymptomatically and were diagnosed by imaging alone [8]. On physical examination, patients may or may not have a palpable abdominal wall defect or bulge in the region of suspected herniation due to the location of the fascial defect deep to the rectus muscle.

Computed tomography remains the imaging modality of choice because it accurately defines the location of the fascial defect, demonstrates the relationship of the herniated bowel to the posterior rectus sheath, and identifies radiographic features concerning for bowel compromise. Findings such as a narrow‐neck fascial defect, bowel wall thickening or hypoenhancement, mesenteric edema, free fluid, proximal bowel dilatation, and a discrete transition point should increase suspicion for incarceration or strangulation and facilitate operative planning [2, 8].

Operative intervention for posterior rectus sheath hernias is typically indicated in patients presenting with complications such as bowel obstruction, strangulation, or persistent symptoms despite conservative measures. Because of the rarity of posterior rectus sheath hernia, no standardized operative guidelines exist. Management should instead follow established principles for incarcerated abdominal wall hernias. Patients with bowel obstruction, strangulation, or suspected ischemia require urgent operative intervention, whereas incidentally discovered asymptomatic defects may be managed electively on an individualized basis. Primary fascial closure has been the most commonly reported repair strategy. Mesh reinforcement may be appropriate for larger defects or poor tissue quality in a clean operative field; however, when bowel resection or contamination is present, many surgeons favor primary tissue repair to minimize the prosthetic infection risk. In the present case, primary repair was selected because bowel perforation and contamination made prosthetic reinforcement less desirable. The favorable postoperative course and absence of recurrence at 1‐year follow‐up support the durability of primary repair in carefully selected contaminated cases.

The recently published systematic review by Magdaleno García et al. [2] summarized the clinical presentation, operative management, and outcomes of reported posterior rectus sheath hernias, highlighting the rarity of strangulation requiring bowel resection. Published reports describing staged management with planned second‐look laparotomy remain exceedingly limited. Our case expands this experience by demonstrating successful staged management with bowel resection, temporary abdominal closure, planned second‐look laparotomy, and delayed definitive repair in a patient without prior abdominal surgery.

## 4. Conclusion

Posterior rectus sheath hernia is a rare but important cause of small bowel obstruction and strangulation that should remain in the differential diagnosis of patients presenting with an interparietal abdominal wall defect on computed tomography, even in the absence of previous abdominal surgery. Recognition of the characteristic retrorectus anatomy, careful interpretation of cross‐sectional imaging, and prompt operative intervention when incarceration or strangulation is suspected are essential to achieving favorable outcomes.

## Funding

No funding was received for this study.

## Conflicts of Interest

The authors declare no conflicts of interest.

## Data Availability

Data sharing is not applicable to this article, as no datasets were generated or analyzed during the current study.
